# Comparison of plastid genomes and ITS of two sister species in *Gentiana* and a discussion on potential threats for the endangered species from hybridization

**DOI:** 10.1186/s12870-023-04088-z

**Published:** 2023-02-20

**Authors:** Jiuyang Mao, Yuze Liang, Xue Wang, Dequan Zhang

**Affiliations:** 1 College of Pharmacy, Dali University, Dali, 671000 Yunnan China; 2 Yunnan Key Laboratory of Screening and Research on Anti-pathogenic Plant Resources from Western Yunnan (Cultivation), Dali, 671000 Yunnan China

**Keywords:** *Gentiana rigescens*, Medicinal plant, Chloroplast genome, Phylogenetic relationship, Genetic introgression

## Abstract

**Background:**

*Gentiana rigescens* Franchet is an endangered medicinal herb from the family Gentianaceae with medicinal values. *Gentiana cephalantha* Franchet is a sister species to *G. rigescens* possessing similar morphology and wider distribution. To explore the phylogeny of the two species and reveal potential hybridization, we adopted next-generation sequencing technology to acquire their complete chloroplast genomes from sympatric and allopatric distributions, as along with Sanger sequencing to produce the nrDNA ITS sequences.

**Results:**

The plastid genomes were highly similar between *G. rigescens* and *G. cephalantha*. The lengths of the genomes ranged from 146,795 to 147,001 bp in *G. rigescens* and from 146,856 to 147,016 bp in *G. cephalantha*. All genomes consisted of 116 genes, including 78 protein-coding genes, 30 tRNA genes, four rRNA genes and four pseudogenes. The total length of the ITS sequence was 626 bp, including six informative sites. Heterozygotes occurred intensively in individuals from sympatric distribution. Phylogenetic analysis was performed based on chloroplast genomes, coding sequences (CDS), hypervariable sequences (HVR), and nrDNA ITS. Analysis based on all the datasets showed that *G. rigescens* and *G. cephalantha* formed a monophyly. The two species were well separated in phylogenetic trees using ITS, except for potential hybrids, but were mixed based on plastid genomes. This study supports that *G. rigescens* and *G. cephalantha* are closely related, but independent species. However, hybridization was confirmed to occur frequently between *G. rigescens* and *G. cephalantha* in sympatric distribution owing to the lack of stable reproductive barriers. Asymmetric introgression, along with hybridization and backcrossing, may probably lead to genetic swamping and even extinction of *G. rigescens*.

**Conclusion:**

*G. rigescens* and *G. cephalantha* are recently diverged species which might not have undergone stable post-zygotic isolation. Though plastid genome shows obvious advantage in exploring phylogenetic relationships of some complicated genera, the intrinsic phylogeny was not revealed because of matrilineal inheritance here; nuclear genomes or regions are hence crucial for uncovering the truth. As an endangered species, *G. rigescens* faces serious threats from both natural hybridization and human activities; therefore, a balance between conservation and utilization of the species is extremely critical in formulating conservation strategies.

**Supplementary Information:**

The online version contains supplementary material available at 10.1186/s12870-023-04088-z.

## Background

*Gentiana* is a famous but extremely complex genus in the family Gentianaceae, comprising approximately 362 species worldwide. Alpine and subalpine regions in southwestern China are the centre of the highest concentration of species in *Gentiana* [1]. *Gentiana rigescens* Franchet is a perennial herb with beautiful flowers and is mainly distributed in Yunnan, Sichuan, and Guizhou [1, 2]. According to Chinese Pharmacopoeia (2020), the species is one of the original species of famous Gentianae Radix et Rhizoma, the so-called “Jianlongdan” [3]. The root and rhizome of the species are traditionally used to clear damp-heat and quench the fire of liver and gall bladder, and have recently been shown to possess neuroprotective and anti-Alzheimer effects[4, 5]. The medicine contains medicinal components, such as iridoids and flavonoids [6, 7]. Owing to over-harvesting and habitat destruction, wild resources of *G. rigescens* have decreased sharply over the last few decades. In addition, the species is narrowly distributed in altitude gradients, probably due to its rigid requirements for biotope, according to our field investigation and a previous study [8]. As a result, *G. rigescens* has been listed in the third class of National Key Protected Wild Medicinal Materials in China [9–11].

**Fig. 1:**
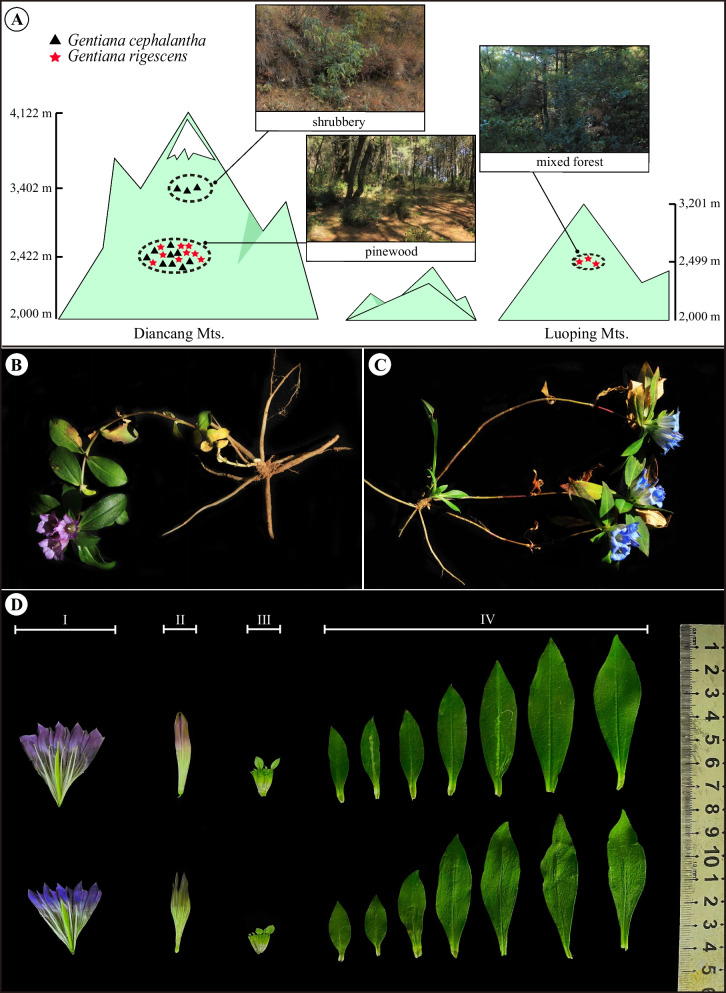
Morphology of *Gentiana rigescens* and *G. cephalantha*, as well as their habitats. (**A**) Habitats of sympatric and allopatric distributions of *G. rigescens* and *G. cephalantha*; (**B**) whole plant of typical *G. rigescens* without rosette leaves; (**C**) whole plant of typical *G. cephalantha* with rosette leaves; (**D**) comparison of floral and leaf morphology between *G. rigescens* (upper) and *G. cephalantha* (under) in the sympatric distribution

*Gentiana cephalantha* Franchet is a sister species to *G. rigescens* with similar morphology, but with better adaptation capacity in diverse habitats (Fig. 1). In Yunnan and adjacent regions, *G. cephalantha* is often used as a medicinal substitute for *G. rigescens* because of its rich resources and confusing morphology [12]. According to Flora of China (1995), both *G. rigescens* and *G. cephalantha* belong to the sect. *Kudoa* [2], but since have been adjusted to sect. *Monopodiae* in the latest taxonomic classification [1]. In terms of morphology, *G. cephalantha* has well-developed rosette leaves and violet corolla, whereas *G. rigescens* does not possess well-developed rosette leaves and carries blue corolla (Fig. 1). Although the two species are widely distributed in SW China with an obvious overlap in geographic ranges, they dominate different altitude gradients. According to a previous field investigation, *G. rigescens* narrowly grows in low elevation areas, but *G. cephalantha* occupies a wide range and diverse habitats in middle and high elevation regions (Table 1). They are generally separated by geography, except for some sympatric distributions. Under such conditions, are *G. rigescens* and *G. cephalantha* conspecific or independent ones in phylogeny? Does nature hybridization occur between the two species (Fig. S1), and will the process pose a threat to the endangered species?

**Table 1:** Information of sample collection for *Gentiana rigescens* and* G. cephalantha*

Species	Number	Locality	Longitude	Latitude	Altitude	Habitats	Voucher specimen	Accession number of cpDNA	Accession number of nrITS
*G.cephalantha* (Allopatric)	GcA01	Diancang Mts., Dali, China	100°06′23.16″E	25°40′56.38″N	3,402 m	shrubbery	ZDQ21038	OM961150	ON820192
	GcA02							OM961165	ON820193
	GcA03							OM961153	ON820194
*G.cephalantha* (Sympatric)	GcS19	Diancang Mts., Dali, China	100°06′50.72″E	25°43′08.25″N	2,422 m	pinewood	ZDQ21048	OM961154	ON820195
	GcS20							OM961164	ON820196
	GcS21							OM961144	ON820197
	GcS22							OM961148	ON820198
	GcS23							OM961160	-
	GcS24							OM961152	ON820200
*G.rigescens* (Sympatric)	GrS25	Diancang Mts., Dali, China	100°06′50.72″E	25°43′08.25″N	2,422 m	pinewood	ZDQ21044	OM961171	ON820204
	GrS26							OM961173	ON820205
	GrS27							OM961168	ON820206
	GrS28							OM961172	ON820207
	GrS29							OM961174	ON820208
	GrS30							OM961169	ON820209
*G.rigescens* (Allopatric)	GrA31	Luoping Mts., Dali, China	99°53′56.11″E	26°01′36.89″N	2,499 m	mixed forest	ZDQ21045	OM961167	ON820201
	GrA32							OM961170	ON820202
	GrA33							OM961175	ON820203

Over the last few decades, molecular techniques have been widely adopted to study species evolution and phylogeny [13, 14], but phylogenetic relationships among *Gentiana* species are not well understood because of the complexity of this genus, insufficient sampling, and low resolution of traditional molecular markers [15–20]. The complete chloroplast genome could provide more useful genetic information than the cpDNA fragments for exploring the phylogeny of complicated genera and discriminating closely related species [21–23]. Dong et al. [24] used plastid genomes to clarify the phylogenetic relationships between 20 complex species in *Lagerstroemia*. Chen et al. [25] also adopted plastid genomes to perform analysis of species discrimination and phylogeny for 21 *Fritillaria* species in China and revealed that the chloroplast genome could efficiently identify closely related species and resolve complicated phylogeny. Ji et al. [26] used complete chloroplast genomes to clarify the phylogenetic relationships among 29 *Paris* species, providing important molecular evidence for clarifying long-standing taxonomic problems in this genus. However, plastid genomes or regions might not really reveal the phylogeny of certain groups due to maternal inheritance; therefore, genetic information from other genomes, such as nrDNA ITS and nuclear genes, could play a vital role here, as well as explore potential hybridization and its threats to endangered species [27, 28].

In the present study, both the plastid genome and ITS were analysed to: (1) compare the basic characteristics of plastid genomes of *G. rigescens* and *G. cephalantha* from sympatric and allopatric distributions; (2) reveal phylogenetic relationships between the two species in *Gentiana*; and (3) explore natural hybridization and genetic introgression between the two species, as well as potential threats to *G. rigescens*. Undoubtedly, this study would be beneficial to understanding the phylogeny of *Gentiana*, as well as the conservation of *G. rigescens* and the pharmacophylogeny of Gentianae Radix et Rhizoma.

## Results

### Characteristics of plastid genomes for *G. rigescens* and *G. cephalantha*

**Fig. 2:**
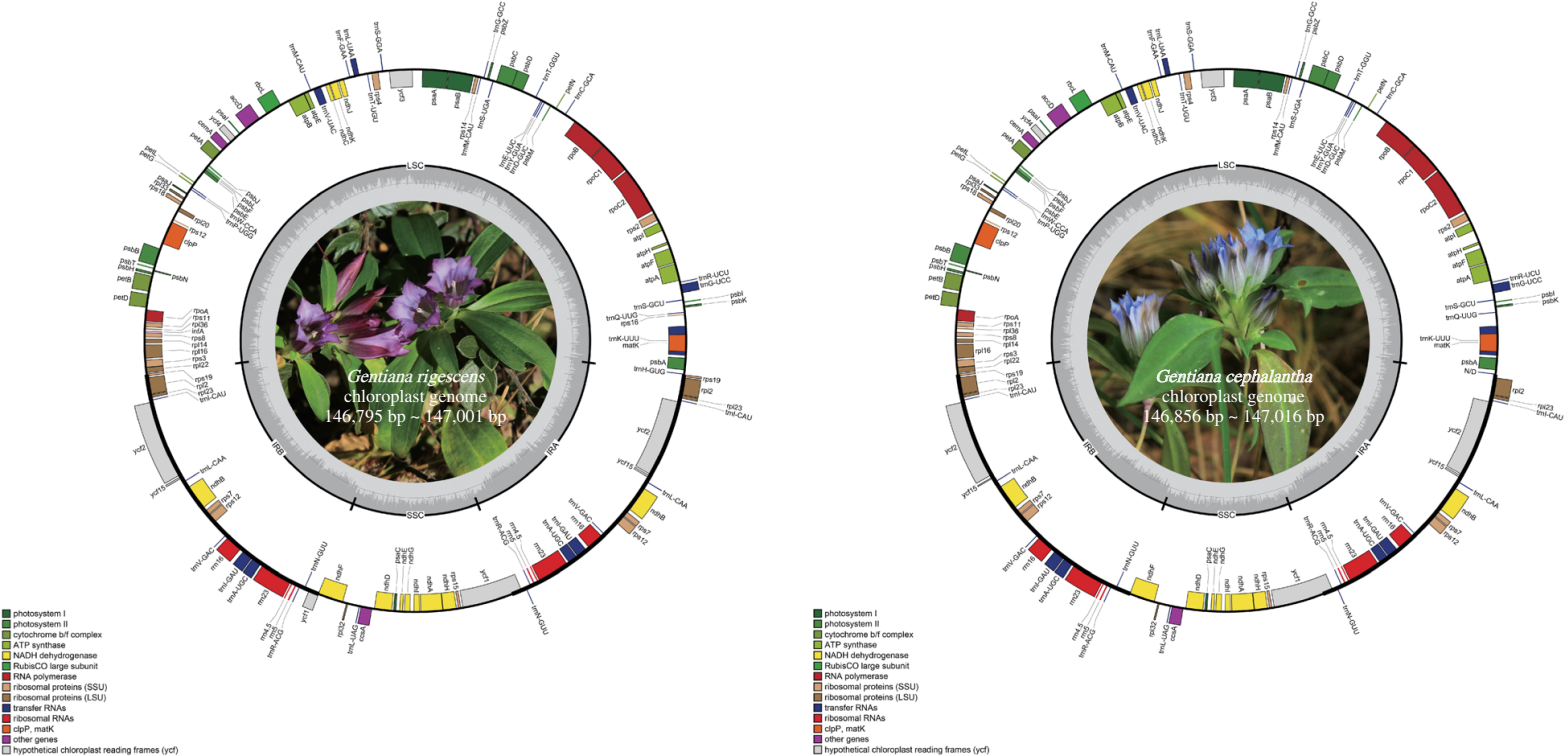
Plastid genome maps of *Gentiana rigescens* and *G. cephalantha*. Genes drawn inside the circle are transcribed clockwise, while those outside the circle are transcribed counter-clockwise. The inner dark gray circle corresponds to GC content and the inner light gray circle corresponds to the AT content. Different colors represent of distinctive genes within separate functional groups

The plastid genomes of *G. rigescens* and *G. cephalantha* were highly similar in their lengths, ranging from 146,795 to 147,001 bp and 146,856 to 147,016 bp, respectively. The intergenic spacer regions (IGS) in *G. cephalantha* were significantly longer than those in *G. rigescens*. Moreover, all chloroplast genomes of the two species had typical circular quadripartite structures, consisting of a large single-copy region (LSC), a small single-copy region (SSC) and a pair of inverted repeat regions (IR) with similar lengths (Table S1, Fig. 2). In total, 116 genes were annotated in these plastid genomes, including 78 protein-coding genes, 30 tRNA genes, four rRNA genes and four pseudogenes (*ψinfA*, *ψycf1*, *ψrps16*, *ψrps19*). Most genes were located in the LSC and SSC regions. Nineteen genes were duplicated in the IR regions, including eight protein-coding genes, seven tRNA genes and four rRNA genes. In addition, 11 protein-coding genes and six tRNA genes were found to contain introns (Table 2).

**Table 2:** Information of the complete chloroplast genomes of *Gentiana rigescens* and* G. cephalantha*

Species	Number	Total (bp)	Large single copy (LSC, bp)	Small single copy (SSC, bp)	Inverted Repeat (IR, bp)	Coding sequence region (CDS, bp)	Intergenic spacer region (IGs, bp)	GC (%)	Total genes	PCG	tRNA	rRNA
*G.cephalantha*	GcA01	147,016	79,439	17,025	25,276	78,444	39,677	37.8	116	78	30	4
	GcA02	147,016	79,439	17,025	25,276	78,444	39,677	37.8	116	78	30	4
	GcA03	147,002	79,385	17,041	25,288	78,444	39,734	37.8	116	78	30	4
	GcS19	147,002	79,385	17,041	25,288	78,444	39,734	37.8	116	78	30	4
	GcS20	146,760	79,373	16,849	25,269	78,492	39,436	37.8	116	78	30	4
	GcS21	146,856	79,353	17,028	25,237	78,492	39,547	37.8	116	78	30	4
	GcS22	146,935	79,352	17,027	25,278	78,492	39,600	37.8	116	78	30	4
	GcS23	146,856	79,353	17,027	25,238	78,492	39,547	37.8	116	78	30	4
	GcS24	146,866	79,364	17,026	25,238	78,492	39,548	37.8	116	78	30	4
*G.rigescens*	GrS25	146,901	79,336	17,027	25,269	78,417	38,479	37.8	116	78	30	4
	GrS26	146,990	79,404	17,026	25,280	78,417	38,552	37.8	116	78	30	4
	GrS27	146,759	79,372	16,849	25,269	78,549	38,198	37.8	116	78	30	4
	GrS28	146,759	79,372	16,849	25,269	78,417	38,198	37.8	116	78	30	4
	GrS29	147,001	79,384	17,041	25,288	78,417	38,580	37.8	116	78	30	4
	GrS30	147,001	79,384	17,041	25,288	78,417	38,580	37.8	116	78	30	4
	GrA31	146,901	79,336	17,027	25,269	78,417	38,479	37.8	116	78	30	4
	GrA32	146,901	79,336	17,027	25,269	78,417	38,479	37.8	116	78	30	4
	GrA33	146,901	79,336	17,027	25,269	78,417	38,479	37.8	116	78	30	4

**Fig. 3:**
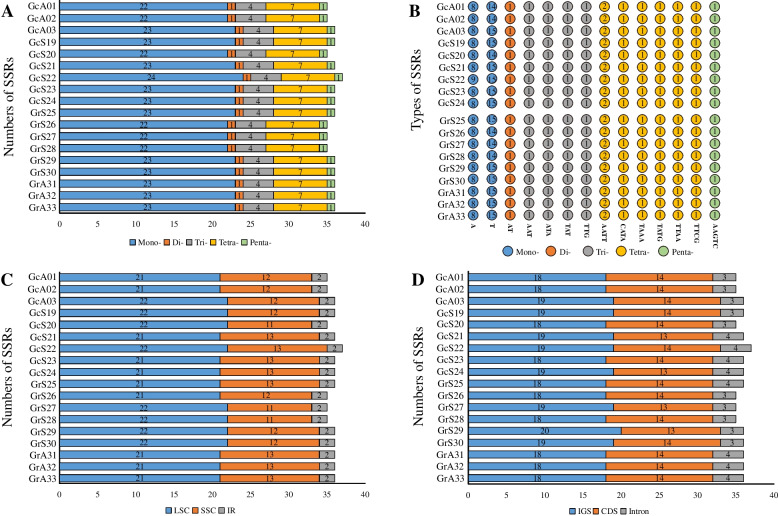
Analysis of simple repeat sequences (SSRs) in complete chloroplast genomes of *Gentiana rigescens* and *G. cephalantha*. (**A**) Numbers of SSRs of five types; (**B**) type of shared SSRs among the eighteen plastid genomes; (**C**) numbers of SSRs in LSC, SSC, and IR regions (IGS), and intronic regions; (**D**) numbers of SSRs in the coding regions (CDS), intergenic spacer regions

Comparative analysis of re-sequencing revealed that the types and numbers of SSRs were highly similar between *G. rigescens* and *G. cephalantha*. This study identified five types of SSRs (mono-, di-, tri-, tetra-, and penta-nucleotides). Among them, mono-nucleotides occurred more frequently in the genomes of the two species (63.6%), followed by tetra-nucleotide repeats (19.6%) and tri-nucleotide repeats (11.3%) (Fig. 3A and B). Most of these SSRs were distributed in the LSC region (60.0%), and a few were distributed in the IR regions (5.6%) (Fig. 3C). In addition, the distribution of SSRs in the intergenic spacer regions (51.8%) was more abundant than that in coding genes (38.6%) or in intronic regions (9.6%) (Fig. 3D).

### Comparison of the plastid genome structures of *G. rigescens* and *G. cephalantha*

**Fig. 4:**
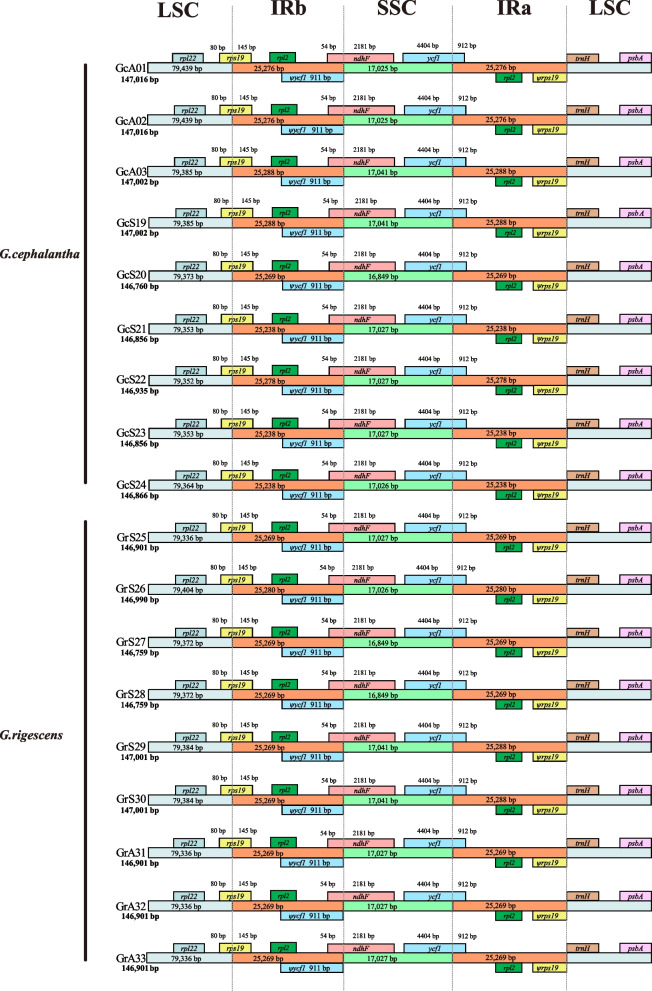
Comparison of LSC, SSC, and IR border regions among the plastid genomes of *Gentiana rigescens* and *G. cephalantha*. Colored boxes for genes represent the gene position

To compare the plastid genome structures of *G. rigescens* and *G. cephalantha*, IR region expansion and contraction were analyzed using IRscope. The results showed that the boundaries of the genomes were highly consistent between the two species. The *rps19* gene had a 145 bp expansion in the IRb region, and the SSC/IRb junction was located in the overlapping region of *ycf1* and *ndhF* genes. In addition, because *ycf1* gene crossed IRa/SSC, most of its sequences were located in the SSC region (4,404 bp), and the remaining sequences were located in IRa (912 bp ), with the same length repetition in IRb (Fig. 4).

**Fig. 5:**
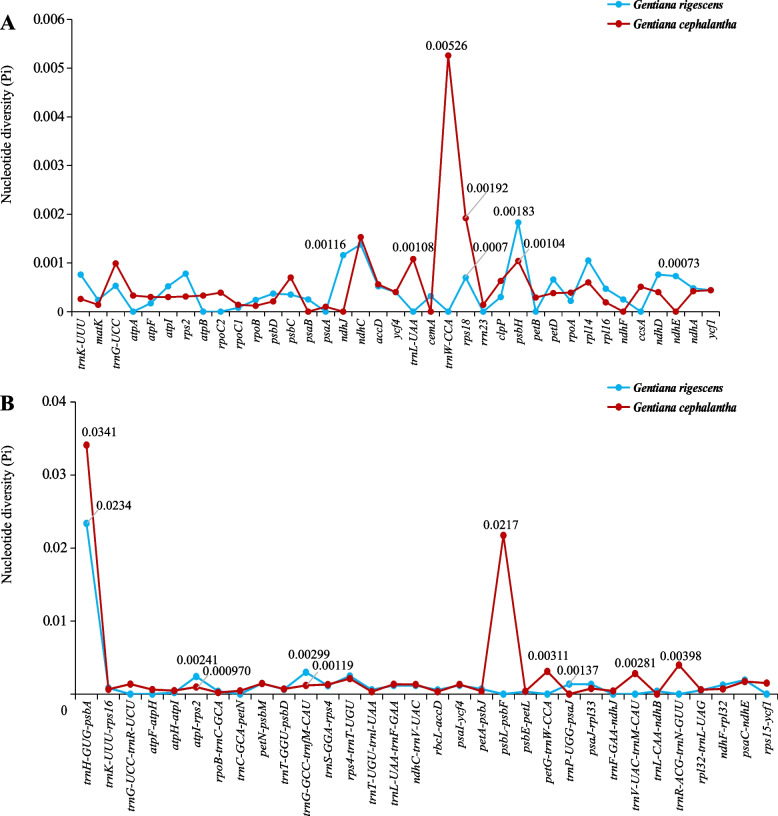
Comparative analysis of nucleotide diversity (Pi) values among the plastid genomes of *Gentiana rigescens* and *G. cephalantha*. (**A**) Nucleotide diversity (Pi) values of coding genes; (**B**) nucleotide diversity (Pi) values of intergenic regions

For the plastid genomes, nucleotide diversity (Pi) of *G. cephalantha* was higher than that of *G. rigescens*. Most of the highly variable sites were concentrated in the LSC and SSC regions. In contrast, the Pi value was relatively low in the IR regions (Table 3). Furthermore, the Pi values were calculated for the coding genes and intergenic spacer regions of the two species. A total of 28 coding genes and 24 intergenic regions in the plastid genomes of *G. rigescens* showed genetic variability within species, as well as 32 coding genes and 30 intergenic regions in those of *G. cephalantha*. Among these, five coding genes and seven intergenic regions possessed high nucleotide diversity (Pi > 0.001; Fig. 5).

**Table 3:** Variable sites in the chloroplast genomes of *Gentiana rigescens* and* G. cephalantha*

	Numbers of sites	Parsimony informative sites	Variable sites	Total numbers of InDel sites	Nucleotide diversity (Pi)
*G. rigescens*					
Genome	147,109	74	89	441	24×10^-6^
LSC	79,460	60	71	171	35×10^-6^
IR	25,299	0	1	30	0.88×10^-6^
SSC	17,051	14	16	210	39×10^-6^
Protein coding genes	78,495	24	31	78	16×10^-6^
*G. cephalantha*					
Genome	147,161	63	123	581	30×10^-6^
LSC	79,501	49	94	240	43×10^-6^
IR	25,304	4	5	66	7×10^-6^
SSC	17,052	6	21	209	36×10^-6^
Protein coding genes	78,492	15	40	48	16×10^-6^

**Fig. 6:**
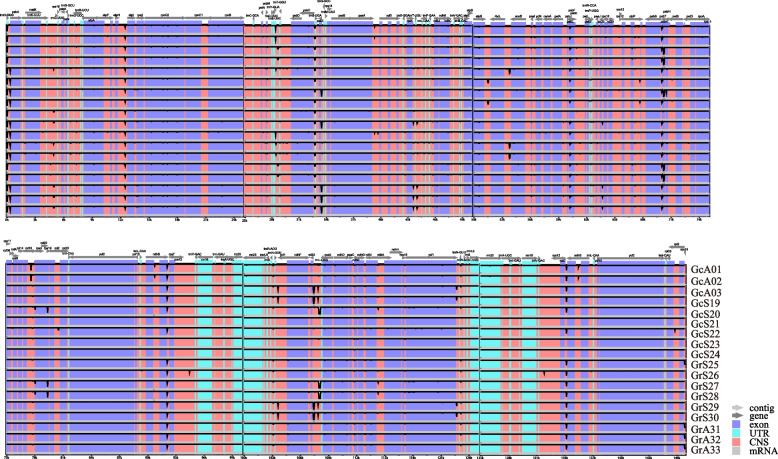
Visualization alignment of the plastid genomes of *Gentiana rigescens* and *G. cephalantha*

The mVISTA analysis showed that the alignments of plastid genomes were highly similar between *G. rigescens* and *G. cephalantha*. Hypervariable regions (HVR) were mainly distributed in the LSC region, but few were distributed in the IR regions. Moreover, the genetic variation of the non-coding regions was greater than that of the coding regions. Therefore, most HVR, including *trnH-GUG-psbA*, *atpH-atpI*, *trnY-GUA-trnT-GGU*, *psbL-psbF*, *psbB-psbT*, *rpl32-trnL-UAG*, and *rps7-ndhB*, were located in the non-coding regions, in addition to *psbT*, *ndhB*, *rpoC2*, and *ycf1* present in the coding regions (Fig. 6).

### Analysis of heterozygotes in ITS sequences

In this study, 18 individuals from *G. rigescens* and *G. cephalantha* were used to acquire ITS sequences by the Sanger sequencing. Subsequently, DNA sequences of good quality from 17 individuals were used for final analysis, except that of GcS23, because of confused overlapping peaks. The total length of the alignment of the ITS sequences was 626 bp, including six informative sites (Table 4). Heterozygotes occurred intensively in individuals from the sympatric distribution, especially in those belonging to *G. rigescens* without rosette leaves. Furthermore, GcS21, GcS24, GrS27, and GrS33 possessed complete heterozygosity across all the variable sites.

**Table 4:** Variable sites of ITS in *Gentiana cephalantha* and *G. rigescens*

Species	Numbers		2	4	4	4	4	Accession number
		5	0	1	1	2	4	
		0	1	7	8	7	9	
*G.cephalantha* (Allopatric)	GcA01	A	C	T	T	A	A	ON820192
	GcA02	A	C	T	T	A	A	ON820193
	GcA03	A	C	T	T	A	A	ON820194
*G.cephalantha* (Sympatric)	GcS19	A	C	T	T	A	A	ON820195
	GcS20	A	C	T	T	A	A	ON820196
	GcS21	R	M	Y	K	W	R	ON820197
	GcS22	A	C	T	T	A	A	ON820198
	GcS24	R	M	Y	K	W	R	ON820200
*G.rigescens* (Sympatric)	GrS25	G	A	C	G	T	G	ON820204
	GrS26	R	M	Y	K	T	R	ON820205
	GrS27	R	M	Y	K	W	R	ON820206
	GrS28	G	A	C	G	T	G	ON820207
	GrS29	G	M	Y	K	T	R	ON820208
	GrS30	G	M	Y	K	T	R	ON820209
*G.rigescens* (Allopatric)	GrA31	G	A	C	G	T	G	ON820201
	GrA32	G	A	C	G	T	G	ON820202
	GrA33	R	M	Y	K	W	R	ON820203

### Phylogenetic analysis of the two species

**Fig. 7:**
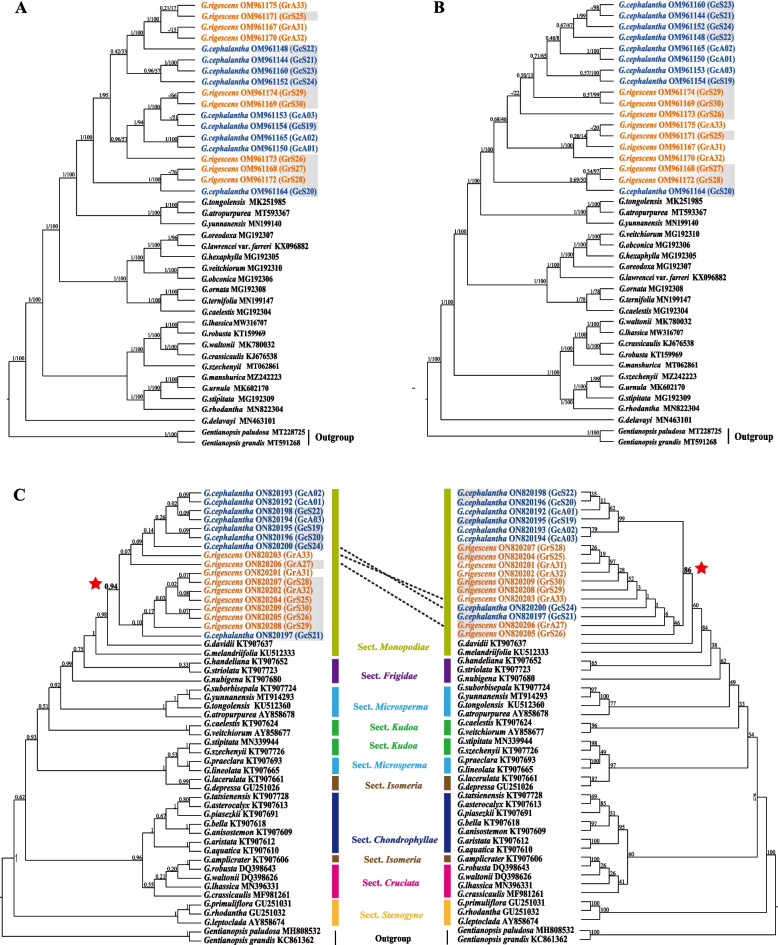
Phylogenetic relationship of *Gentiana rigescens* and *G. cephalantha*. (**A**) complete chloroplast genomes; (**B**) CDS regions; (**C**) BI (left) and ML (right) phylogeny based on the ITS sequences. Number above nodes are support values with BI posterior probabilities (PP) values on the left and ML bootstrap (BS) values on the right. Orange fonts represent *G. rigescens*, dark blue fonts represent *G. cephalantha*, and grey shades indicate the individuals located in the sympatric distribution

To explore the phylogenetic relationship between *G. rigescens* and *G. cephalantha*, maximum likelihood (ML) and Bayesian inference (BI) were mainly performed based on chloroplast genomes, coding sequences (CDS), and ITS sequences; and then phylogenetic analysis using representative HVR was also carried out. The results showed that topological structures between the ML and BI trees were highly identical for plastid datasets and showed little inconformity for the ITS samples (Fig. 7), but they were obviously inconsistent for the HVR sequences (Fig. S2). However, there were significant differences among the trees based on plastid genomes, CDS, and ITS sequences. For all the three datasets, both *G. rigescens* and *G. cephalantha* were clustered into a monophyletic branch; however, the phylogeny between the chloroplast genomes and ITS sequences of the two species were completely different (Fig. 7). Individuals of *G. rigescens* and *G. cephalantha* were intermingled in the trees of plastid genomes, as well as those of HVR sequences, but could be weakly distinguished by the CDS dataset. On the contrary, the phylogenetic trees based on ITS sequences showed that the two species were well discriminated, except for the potential hybrids, namely GcS21, GrS27, and GrS33 in BI, as well as GcS21 and GcS24 in the ML tree. Moreover, the ML and BI analysis using ITS sequences covering nine sections of *Gentiana* (14 sect.) supported the independence of sect. *Monopodiae* separated from sect. *Kudoa*.

## Discussion

### Comparison on the plastid genomes between *G. rigescens* and *G. cephalantha*

In this study, the structures of the plastid genomes between *G. rigescens* and *G. cephalantha* were highly similar. The total lengths of the genomes were from 146,759 bp to 147,001 bp in *G. rigescens* and from 146,856 bp to 147,016 bp in *G. cephalantha*, which were shorter than those of most of the other species in *Gentiana*, as well as other genera in Gentianaceae [29, 30]. Four common pseudogenes were found in the plastid genomes of *G. rigescens* and *G. cephalantha*. Among them, the existence of *ψycf1* and *ψrps19* could be attributed to boundary effects, whereas *ψinfA* and *ψrps16* are probably caused by gene transfer and loss during evolution, which are also common in species of Gentianaceae and other family [29, 31–35]. In addition, deletion of *ndh* gene has been frequently reported in *Gentiana*, but we could not detect it in either of the two species [29].

Simple sequence repeats (SSRs) are useful tools for evaluating population genetic diversity and structure of species and are thus widely adopted in studies on the conservation of endangered species and evolution of complicated groups [36, 37]. The number of SSRs was 35 – 37 in the two species, and mono-nucleotide repeats were the most abundant, especially A/T repeat units. These results were consistent with those of our previous study [30]. In addition, most of the SSRs were distributed in the intergenic spacer region, followed by the coding genes (*rpoC1*, *psaB*, *atpB*, *ndhF*, *ycf1*), but the least in the intronic region, which was also similar to those reported for other *Gentiana* species [35].

Hypervariable regions (HVR) not only resolve phylogeny and identify species at the species level but also provide critical information for exploring species differentiation and genetic structure at the population level [38]. The results of mVISTA and the sliding window have been used for screening the HVR [39, 40]. Herein, we detected eight HVR from non-coding (*trnH-GUG-psbA*, *atpH-atpI*, *petG-trnW-CCA*, *rpl32-trnL-UAG*, *rps7-ndhB*) and coding regions (*ndhB*, *rpoC1*, *psbH*), which have been widely adopted for studies on phylogenetic analysis and DNA barcodes in angiosperms [25, 41–45]. In contrast, *matK* and *rbcL* as core DNA barcodes showed no genetic variation in *G. rigescens* and *G. cephalantha*, further supporting the results of our previous study on DNA barcodes in *Gentiana* [19]*.* It should be noted that the *trnH-psbA*, as an efficient barcode for identifying species, showed the highest Pi value in the sliding window. A possible reason is that *Gentiana* species are prone to base inversion in this region, leading to overestimation of genetic variation within the species [46]. Therefore, this region was deemed unsuitable to identify *Gentiana* species, as it has been reported before [19, 20]. Expansion and contraction of the IR regions are important factors leading to changes in plastid genome size and play an important role in the stability and evolution of the genome structure [47–49]. The results showed that the genetic compositions of the four junctions in the chloroplast genomes were highly identical between the two species, similar to the findings from studies on other species belonging to the Gentianaceae [29, 31]. The expansion and contraction of *rps19*, *ndhF*, and *ycf1* located at the boundaries of plastid genomes were consistent between *G. rigescens* and *G. cephalantha*, probably due to conservatism during plastid evolution of *Gentiana* [30].

### Phylogenetic analysis and species definition of *G. rigescens* and *G. cephalantha*

Currently, plastid genomes have been widely used to reveal the phylogeny of complicated genera or closely related species and developed as field known as phylogenomics [24, 26, 50]. *Gentiana* is an extremely complicated genus in Gentianaceae with approximately 362 species worldwide, including a series of confusing species such as *G. rigescens* and *G. cephalantha* [2]. Although the two species can be discriminated by the basal rosettes of *G. cephalantha* and its blue corolla, they are still easily confused because of their similar but variable morphology, especially in sympatric distributions [51]. In our study, the two species were clustered into a monophyletic group using plastid genomes, CDS, and ITS datasets, which showed that the two species were closely related in terms of phylogeny. According to the ML and BI analysis, complete chloroplast genomes, coding sequences, and HVR regions could not correctly discriminate individuals from *G. rigescens* and *G. cephalantha*; on the contrary, ITS could gather the two species into distinct independent clades after removal of potential hybrids (Fig. 7). First, in the phylogenetic tree based on the plastid genomes, all individuals were heavily mixed which was also supported by comparative analysis of the chloroplast genomes of the two *Gentiana* species. Comparatively, the tree constructed using CDS showed better resolution, in which most individuals of *G. cephalantha* (except GcS20) formed a monophyletic group. The present result also supports that from the previous report in which protein-coding sequences were adopted to reveal phylogenetic relationships of species in *Gentiana* sect. *Kudoa* and evaluate divergence times [45]. On the contrary, *G. rigescens* and *G. cephalantha* were clustered into two independent clades based on ITS sequences, regardless of the possible hybrids (Fig. 7). Therefore, both *G. cephalantha* and *G. rigescens* are closely related but independent species, according to their morphological and molecular evidences. The present results further verified the value of ITS as shown in previous phylogenetic analyses of *Gentiana* species [52]. Moreover, molecular evidence from the current study also supported the adjustment of the two species into sect. *Monopodiae* from sect. *Kudoa* [1, 2, 16].

It is well known that the chloroplast genome could provide much richer genetic information and a better solution than DNA regions for revealing the phylogeny of complicated genera and discriminating closely related species [53, 54]. In our previous study, the plastid genome was confirmed to be a DNA super barcode that could efficiently discriminate most species in *Fritillaria* in China, but the results from ITS and other universal DNA barcodes analyses were disappointing [25, 53]. Zhang et al. [55] adopted plastid phylogenomics to reveal the deep phylogenetic relationships and diversification history of Rosaceae. Li et al. [56] also reported that phylogenomic analyses of *Fagopyrum* supported the division of the cymosum and urophyllum groups, and resolved the systematic position of subclades within the urophyllum group. However, it should be noted that plastid genomes could enhance species discrimination and reveal phylogeny, but they are still not powerful enough to resolve all species in complicated genera and recently diverged lineages, such as *Paris*, *Berberis*, and *Rhododendron* [26, 57, 58]. This research provides a case study in which only the plastid genome resulted in incorrect assessment of phylogenetic relationships and species discrimination; therefore, nuclear genes should be adopted alongside plastid genomes to reveal intrinsic phylogeny and trace species boundaries.

### Species evolution in *Gentiana* and potential hybridization between *G. rigescens* and *G. cephalantha*

According to previous studies, both *G. rigescens* and *G. cephalantha* are widely distributed in Yunnan, Sichuan, Guizhou and adjacent regions with obvious geographical overlap [1, 2]. Field investigations revealed that the two species are generally distributed in allopatric regions. *G. rigescens* narrowly grows at lower altitudes, but *G. cephalantha* can occur in a much wider range at higher altitude gradients (Fig1). It is well known that the Qinghai-Tibet Plateau (QTP) and its adjacent mountains are key regions for evolution of alpine plants, and *Gentiana* was revealed to originate from QTP in the Eocene and then spread to other distributions from the late Miocene onwards, a phenomenon named as the "out of Tibet" hypothesis [59]. According to molecular clock dating using pollen and seed fossils, divergence time of *Gentiana* was deduced to be 29 million years, and the divergence of *G. rigescens* and *G. cephalantha* happened about 0.51 Ma and supposedly is the youngest node of differentiation. Obviously, *G. cephalantha* is more adaptable to the cooling climate and diverse habitats, so it colonizes a much broader territory than *G. rigescens* in altitude gradients (Fig. 1). The two species generally grow in allopatric areas at altitudes, except for a few sympatric distributions that are generally located in the border zones between *G. rigescens* and *G. cephalantha*. In the present study, potential hybridization was observed in sympatric distributions in the Diancang Mountains, which showed a visible intermediate corolla color compared to typical *G. rigescens* and *G. cephalantha* (Fig. S1); meanwhile, possible hybrids were preliminarily identified based on the ITS sequences (Table 4). Natural hybrids between *Gentiana stramine*a and *G. siphonantha* have been confirmed based on molecular evidence [60]. Considering the overlapping of flowering, geographic isolation might be the main factor for the two species, but possible physiological factors should be explored using pollination biology and other evidences [61].

### Threats to *G. rigescens* from hybridization and the balance between conservation and utilization of the endangered species

Natural hybridization occurs widely among species and evolutionary lineages in plants and plays critical role in species evolution [62, 63]. Hybridization probably leads to phenotypic novelty and results in the formation of new species [64]. Meanwhile, the same process may cause the breakdown of species integrity and ultimately drive rare species to extinction through genetic swamping, where the rare form is replaced by hybrids or demographic swamping [63, 65]. *G. rigescens*, a rare species, is threatened by hybridization with its common congeneric species. According to the ITS sequences of the 17 individuals of the two species (Table 4 and Fig. 7), hybridization was massively detected in the sympatric distribution and sporadically in the territory of *G. rigescens*, but seemingly not in the allopatric distribution of *G. cephalantha*. Therefore, asymmetric introgression had a significantly more serious impact on *G. rigescens* than *G. cephalantha*. Hybridization, as well as subsequent backcrossing between hybrids and the common species, constantly dilutes the genetic loci of the rare one [65–67], which finally results in genetic erosion of *G. rigescens*. As two sister species, no steady reproductive barrier exists between *G. rigescens* and *G. cephalantha,* in addition to geographic isolation along altitude gradients. Habitat change along with human activities provides more possibilities for hybridization between the newly divergent species and affords new niches to hybrids [63]. As a result, the rare species may be replaced by hybrids, even faced with extinction due to genetic swamping or demographic swamping from hybridization [63, 66–68].

As an endangered species possessing medicinal value, balance between efficient conservation and rational utilization of *G. rigescens* is an urgent problem to be resolved. First, strict conservation of the species should be adopted for basic measurements, so as to avoid serious harvesting and destruction. Then, exploring new original species of Gentianae Radix et Rhizoma by pharmacophylogeny could be an efficient method to satisfy medicinal demands, such as *G. cephalantha* [69]. Although both species are used as traditional Chinese medicine “Jianlongdan” in Yunnan and adjacent regions, further evaluation of the medicinal quality and efficacy of medicines between the two species is necessary. Moreover, developing artificial cultivation for *G. rigescens* would be beneficial for balancing conservation and utilization. It should be noted that screening pure individuals without genetic pollution is critically important to ensure the quality of the medicine in consideration of hybridization and genetic introgression. Finally, protecting habitat diversity could provide different niches for *G. rigescens* and *G. cephalantha*, and geographic isolation might be an efficient reproductive barrier to maintaining the independence of the species [66, 68].

## Conclusion

In this study, the next-generation sequencing and Sanger sequencing were used to investigate plastid genomes and ITS sequences of *G. rigescens* and *G. cephalantha* from sympatric and allopatric distributions. Comparative analysis of the plastid genomes showed that the two *Gentiana* species possess highly similar genome structures. *G. rigescens* and *G. cephalantha* were clustered into a monophyletic group during phylogenetic analyses based on the plastid genome, CDS, and ITS regions, supporting their close relationship. Moreover, the two species were mixed in the trees constructed using plastid genomes, CDS, and HVR, but they could form obvious monophyly in phylogenetic analysis based on ITS, excluding the potential hybrids. The present study supports the current treatment of *G. rigescens* and *G. cephalantha* as independent but closely related species in *Gentiana* based on the present morphological and molecular evidences. Natural hybridization occurs intensively between the two species, especially in sympatric distribution. Asymmetric introgression has a significantly more serious impact on *G. rigescens* than *G. cephalantha*, and this process probably leads to constant dilution of genetic loci and even extinction of the rare species. Conserving wild resources, exploring alternative species, developing artificial cultivation, and maintaining habitat diversity are efficient measurements for ensuring a balance between conservation and utilization. In summary, the complete chloroplast genome possesses rich genetic information; thus, it can be used to explore the phylogenetic relationships of closely related species or complicated genera. However, phylogenetic trees might not reveal real phylogeny when we only use plastid genomes or genes because of matrilineal inheritance, and datasets from nuclear genomes or regions are crucial for exploring the truth.

## Methods

### Materials collection

Dali (Tali) in China is the origin of the type specimens of *G. rigescens* and *G. cephalantha*. In the present study, fresh and clean leaves of adult individuals were collected from the Diancang and Luoping Mountians in Dali, China (Fig. 1 and Table 1). The leaves were directly dried using allochroic silica gel during fieldwork and were used as molecular materials. Herein, we sampled six accessions of each species from their sympatric distribution in the Diancang Mountians (2,422 m). Considering the morphological similarity between the two species, rosette leaves and blue corolla were regarded as distinctive features for *G. cephalantha* that was discriminated from *G. rigescens* (Fig. 1). Three individuals of *G. cephalantha* were also sampled from an allopatric distribution of alpine shrubbery in the alpine region of the same mountain (3,402m), and three individuals representing *G. rigescens* were collected from one allopatric distribution in the Luoping Mountains (Fig. 1). Eight individuals representing the two species were used for DNA sequencing. To avoid sampling individuals from the same female parent, geographic distances among individuals were above 30 m. Meanwhile, 2-3 mature individuals with flowers from each site were excavated and used as the voucher specimens (Table 1). The collection of molecular materials and specimens was approved by the Forestry and Grassland Administration in Dali Prefecture, China (Grant no. 2021-137). All specimens were identified by Professor Dequan Zhang according to Ho’s classification system (1995) and Flora of China (2001) [1, 2] and then deposited at the Herbarium of Medicinal Plants and Crude Drugs of the College of Pharmacy, Dali University.

### Molecular experiments

Total genomic DNA was extracted from the dried leaves using a modified CTAB method [70]. DNA quality and concentration were detected using 1.2% agarose gel electrophoresis and spectrophotometer (Bio-Rad, Hercules, CA, USA). The DNA was then sheared to yield approximately 500 bp long fragments for library construction. The library was sequenced on an Illumina Hiseq 2500 platform according to the standard protocol of manufacturer’s instructions. About 2-4 Gb raw paired-end reads (2×150 bp) were obtained for each individual of the two species. Next-generation sequencing were performed by Novogene Bioinformatics Technology Co. Ltd., Beijing, China.

The amplification reaction system of ITS sequence was: 2×Taq PCR MasterMix (10 μl), two ITS primers (0.3 μl), template DNA (1 μl) and ddH_2_O (8.4 μl). The PCR amplification procedure was as follows: pre-denaturation at 94°C for 2 min; 94°C for 40 s, 55°C for 45 s, and 72°C for 55 s, after 35 cycles; 72°C extension for 7 min primer sequence: ITS4: 5'-TCC TCC GCT TAT TGA TAT GC-3'; ITS5: 5'-GGA AGT AAA AGT CGT AAC AAG G-3'. The entire ITS sequence was sequenced by the Kunming Institute of Botany, Chinese Academy of Sciences.

### Assembly, annotation, and submission of plastid genomes and ITS sequence

Raw data were filtered using Trimmomatic v.0.32 with default settings [71], and paired-end reads in the clean data were assembled into contigs using GetOrganelle.py [72]. After assembly, the *de novo* assembly graphs were visualized and edited using Bandage, and then a whole or nearly whole circular plastid genome was generated [73]. Using *G. rigescens* (MT062862) downloaded from the National Center for Biotechnology Information (NCBI, https://www.ncbi.nlm.nih.gov/) as the reference sequence, MAFFT was performed in the Geneious v.11.1.4 [74, 75], and annotation, modification and manual correction were performed. Circular genome visualization was generated using the online program OGDRAW (https://chlorobox.mpimp-golm.mpg.de/OGDraw.html).

Original ITS DNA sequences were assembled using the SeqMan program (DNASTAR, Lasergene) [76] and then aligned in MEGA v.7.0.26 [77]. The boundaries of ITS1, 5.8S, and ITS2 were determined using the reference sequence of *G. cephalantha* (KT907627) from NCBI. All annotated plastid genomes and ITS sequences were confirmed, edited using Sequin software, and submitted to the GenBank database (Table 1).

### Comparative analysis of the plastid genomes

Using the website IRscope (https://irscope.shinyapps.io/irapp/), the IR boundaries of 18 plastid genomes of *G. rigescens* and *G. cephalantha* were analyzed [78], followed by manual editing. The lengths of large single copy (LSC), small single copy (SSC), inverted repeats (IRs), protein-coding genes, and intergenic spacer regions for each of genomes were counted by Geneious v.11.1.4. The online program mVISTA (https://genome.lbl.gov/vista/mvista/instructions.shtml) was used for sequence alignment and variation analysis of plastid genomes in Shuffle-LAGAN mode [79, 80], with the annotation of *G. cephalantha* (MN199135) as a reference. In addition, parsimony informative sites and variable sites were analyzed, and a sliding window was used to analyze the nucleotide diversity (Pi) of complete chloroplast genomes and protein-coding genes in *G. rigescens* and *G. cephalantha* by DnaSP v.6.11 (with a window length of 600 bp and a step size of 200 bp) [81]. MISA was adopted to evaluate SSRs in plastid genomes, among which the SSRs parameters of mono-, di-, tri-, tetra-, penta-, and hexa-nucleotide motifs were set as 10, 5, 4, 3, 3, and 3 [82].

### Phylogenetic analysis for *G. rigescens *and* G. cephalantha*

A total of 18 new plastid genomes from *G. rigescens* and *G. cephalantha*, and 23 published plastid genomes for other *Gentiana* species from NCBI were used for phylogenetic analysis (Table S2). In addition, phylogenetic trees were constructed based on the 17 new ITS sequences (excluding GcS23) and 32 reported ITS sequences from other *Gentiana* species (Table S3). Two *Gentianopsis* species were selected as outgroups for constructing phylogenetic trees based on the plastid genomes, CDS, and ITS datasets; moreover, phylogenetic analysis using HVR was additionally performed for the two species. The most appropriated model of sequence substitution for plastid genomes (GTR + G + I), CDS (GTR + G + I), HVR (GTR or GTR+G), and ITS (GTR + G + I) were screened by MEGA v.7.0.26 [78]. Phylogenetic analysis was performed using Maximum likelihood (ML) and Bayesian inference (BI). ML analysis was performed using the RAxML v.8.2.10. The local bootstrap (BS) probability of each branch was calculated with 1,000 repetitions [83]. BI analysis was performed using MrBayes v.3.2.6 [84]. The Markov Chain Monte Carlo (MCMC) algorithm was calculated for 1000,000 generations with a sampling of trees every 1,000 generations. The first 25% of the generations were discarded as burn-in, and posterior probability (PP) values were determined from the remaining trees to evaluate the support rate of each branch. The state was considered to have been reached when the average standard deviation of the split frequency was < 0.01. Finally, all the methods were performed in accordance with relevant guidelines and regulations.

## Supplementary Information

**Additional file 1: Figure S1.** Phenotype of potential hybrids between *Gentiana rigescens* and *G. cephalantha*.

**Additional file 2: Figure S2.** Phylogenetic relationships of *Gentiana rigescens* and *G. cephalantha* based on each HVR regions.

**Additional file 3: Table S1.** Gene contents of the plastid genomes of *Gentiana rigescens* and *G. cephalantha*.

**Additional file 4: Table S2.** The complete chloroplast genomes of *Gentiana* species downloaded from NCBI.

**Additional file 5: Table S3.** The nrITS sequences of *Gentiana* species downloaded from NCBI.

## Data Availability

All sequences (plastid genomes and ITS sequences) used in this study have been submitted to the National Center for Biotechnology Information (NCBI, https://www.ncbi.nlm.nih.gov/) with accession numbers (OM961144, OM961148, OM961150, OM9652-OM961154, OM961160, OM961164, OM961165, and OM961167-OM961175 for plastid genomes; ON820192-ON820203 for ITS sequences) (Table 1). All the sequences will be available after publication of this manuscript.
