# Supplementary figures and images for: Comparison of plastid genomes and ITS of two sister species in Gentiana and a discussion on potential threats for the endangered species from hybridization
Source: BMC Plant Biol. 2023 Feb 20;23:101. doi: 10.1186/s12870-023-04088-z (PMC9940437; doi:10.1186/s12870-023-04088-z)

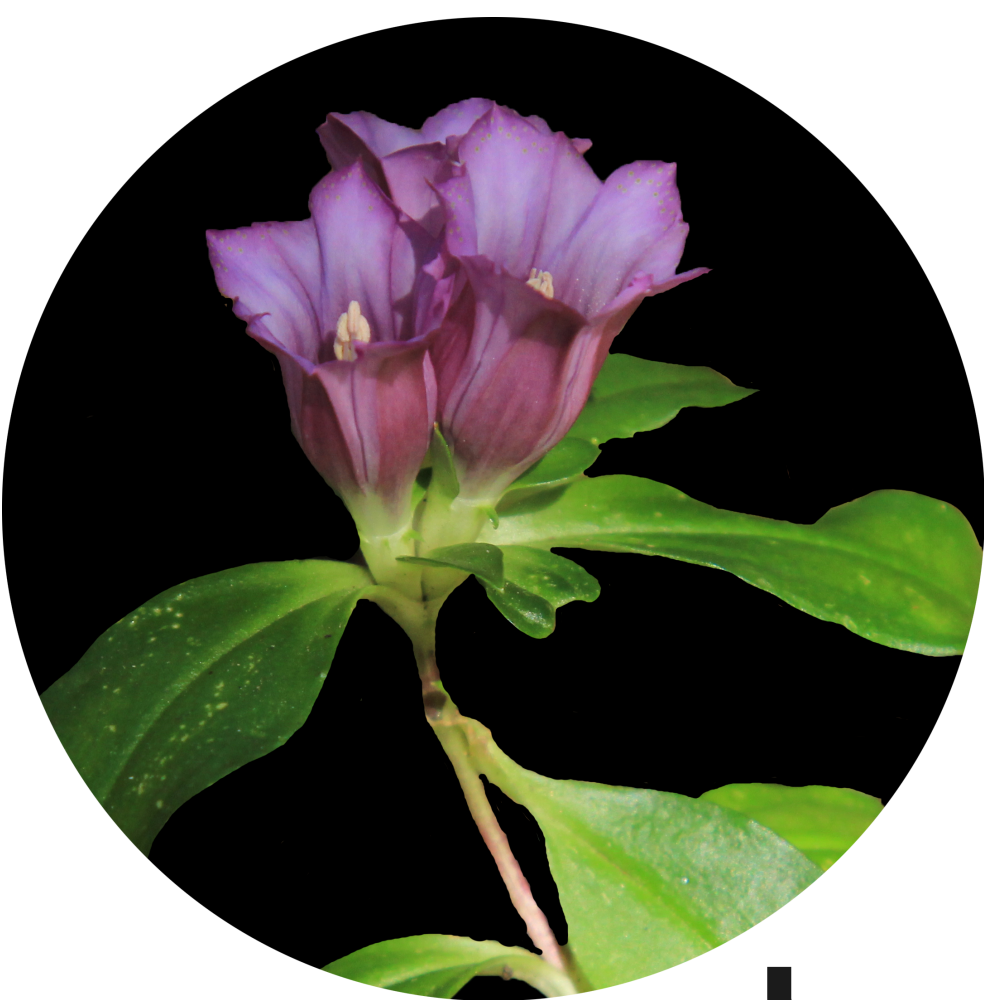

*Gentiana rigescens*

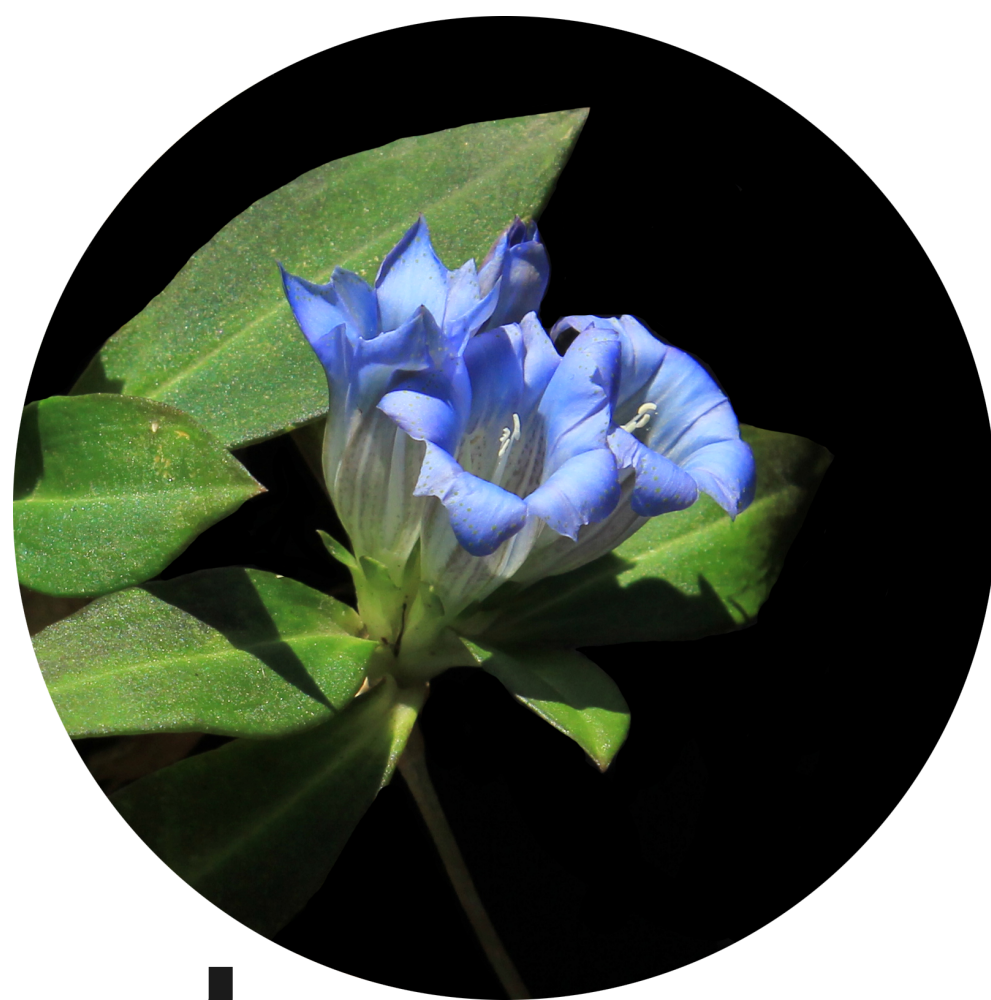

*Gentiana cephalantha*

Hybrid?

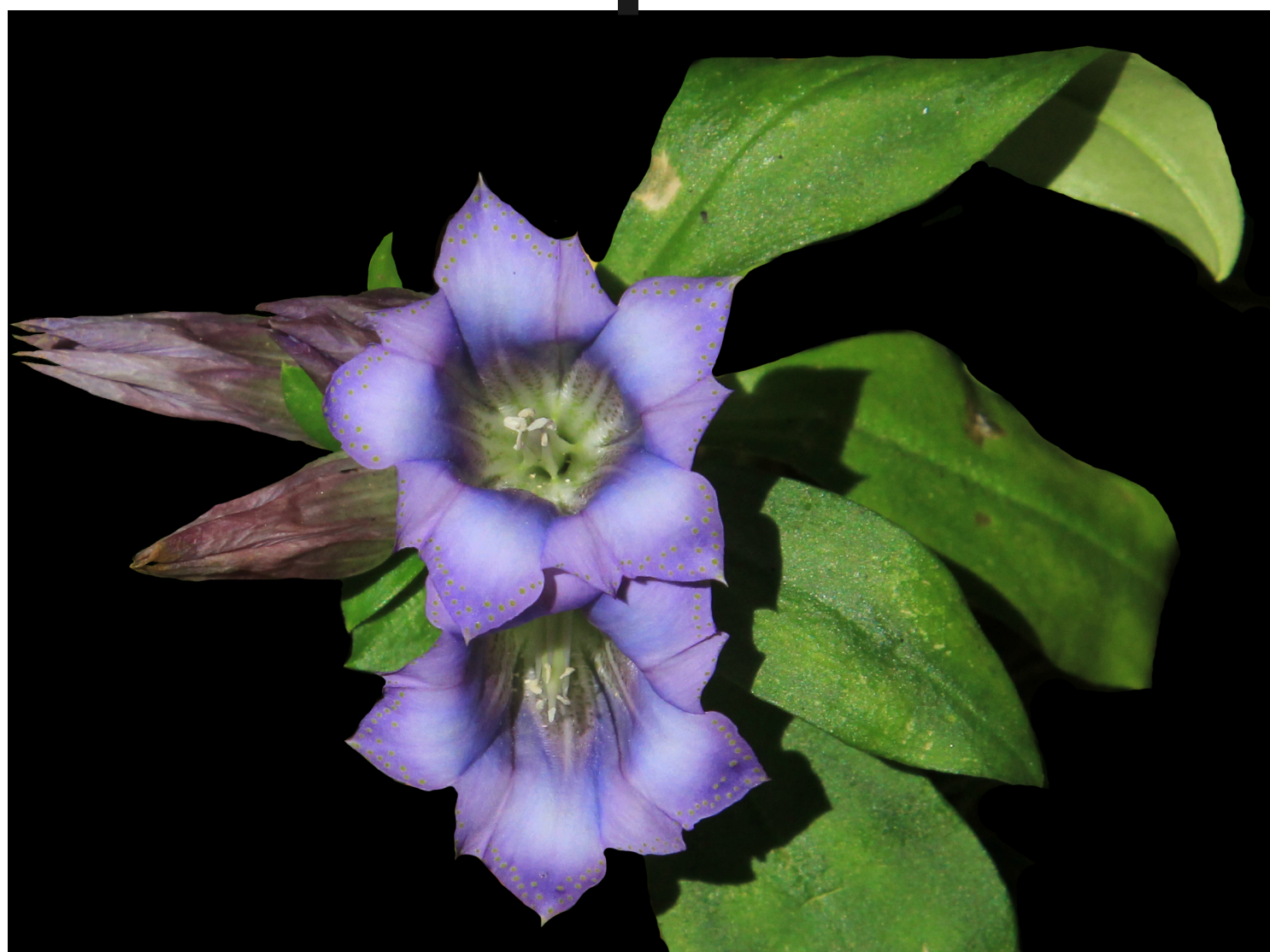

Potential intermediate

Supplement: Supplementary file 1 — Additional file 1: Figure S1. Phenotype of potential hybrids between Gentiana rigescens and G. cephalantha. [file 12870_2023_4088_MOESM1_ESM.pdf]

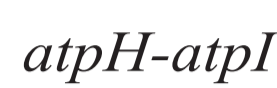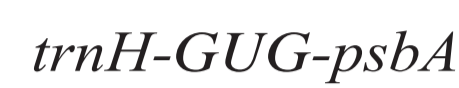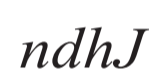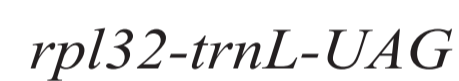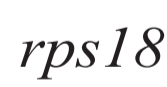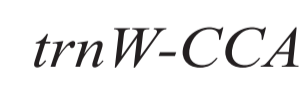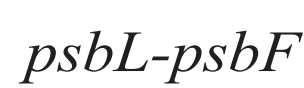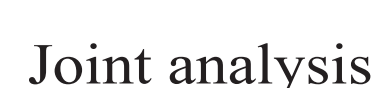

Supplement: Supplementary file 2 — Additional file 2: Figure S2. Phylogenetic relationships of Gentiana rigescens and G. cephalantha based on each HVR regions. [file 12870_2023_4088_MOESM2_ESM.pdf]
