# Supplementary material for: Comparison of plastid genomes and ITS of two sister species in Gentiana and a discussion on potential threats for the endangered species from hybridization
Source: BMC Plant Biol. 2023 Feb 20;23:101. doi: 10.1186/s12870-023-04088-z (PMC9940437; doi:10.1186/s12870-023-04088-z)
Supplement: Supplementary file 3 — Additional file 3: Table S1. Gene contents of the plastid genomes of Gentiana rigescens and G. cephalantha. [file 12870_2023_4088_MOESM3_ESM.docx]

| **Table S1** Gene contents of the plastid genomes of *Gentiana rigescens* and *G. cephalantha* | | |
| --- | --- | --- |
| Gene group | Gene function | Gene name |
| Self-replication | Large subunit of ribosome | *rpl2*^I*^*, rpl14, rpl16*^*^*, rpl20, rpl22, rpl23*^I^*, rpl32, rpl33, rpl36* |
|  | Small subunit of ribosome | *rps2, rps3, rps4, rps7*^I^*, rps8, rps11, rps12*^I*^*, rps14, rps15, ψrps16, rps18, ψrps19*^I^ |
|  | RNA polymerase | *rpoA, rpoB, rpoC1^*^, rpoC2* |
|  | rRNA | *rrn4.5*^I^*, rrn5*^I^*, rrn16*^I^*, rrn23*^I^ |
|  | tRNA | *trnA-UGC*^I*^*, trnC-GCA, trnD-GUC, trnE-UUC, trnF-GAA, trnG-GCC, trnG-UCC*^*^*, trnH-GUG, trnI-CAU*^I^*, trnI-GAU*^I*^*, trnK-UUU*^*^*, trnL-CAA*^I^*, trnL-UAA*^*^*, trnL-UAG, trnM-CAU, trnfM-CAU, trnN-GUU*^I^*, trnP-UGG, trnQ-UUG, trnR-ACG*^I^*, trnR-UCU, trnS-GCU, trnS-GGA, trnS-UGA, trnT-GGU, trnT-UGU, trnV-GAC*^I^*, trnV-UAC*^*^*, trnW-CAA, trnY-GUA* |
| Photosynthesis | Photosystem I  Photosystem II  NADH dehydrogenase  Cytochrome b/f complex  ATP synthase  Large subunit of rubisco | *psaA, psaB, psaC, psaI, psaJ*  *psbA, psbB, psbC, psbD, psbE, psbF, psbH, psbI, psbJ, psbK, psbL, psbM, psbN, psbT, psbZ*  *ndhA*^*^*, ndhB^I^, ndhC, ndhD, ndhE, ndhF, ndhG, ndhH, ndhI, ndhJ, ndhK*  *petA, petB*^*^*, petD*^*^*, petG, petL, petN*  *atpA, atpB, atpE, atpF*^*^*, atpH*^*^*, atpI*  *rbcL* |
| Other genes | Translational initation factor  Maturase  Protease  Envelope membrane protein  Subunit of Acetyl-carboxylase  C-type cytochrome synthesis  Open reading frames | *ψinfA*  *matK*  *clpP*^*^  *cemA*  *accD*  *ccsA*  *ψycf1*^#^*, ycf2*^I^*, ycf3*^*^*, ycf4, ycf15*^I^ |
| Note:"I" indicates that the gene is located in the IR regions, "*" indicates that the gene contains introns, and "*ψ*" represents a pseudogene. | | |

[
