# Supplementary material for: Comparison of plastid genomes and ITS of two sister species in Gentiana and a discussion on potential threats for the endangered species from hybridization
Source: BMC Plant Biol. 2023 Feb 20;23:101. doi: 10.1186/s12870-023-04088-z (PMC9940437; doi:10.1186/s12870-023-04088-z)
Supplement: Supplementary file 4 — Additional file 4: Table S2. The complete chloroplast genomes of Gentiana species downloaded from NCBI. [file 12870_2023_4088_MOESM4_ESM.docx]

| **Table S2** The complete chloroplast genomes of *Gentiana* species downloaded from NCBI | | | |
| --- | --- | --- | --- |
| No. | GenBank accession number | Species | Sequence length (bp) |
| 1 | MK251985 | *Gentiana tongolensis* | 145,757 |
| 2 | MT593367 | *Gentiana atropurpurea* | 145,757 |
| 3 | MN199147 | *Gentiana ternifolia* | 137,516 |
| 4 | MG192308 | *Gentiana ornata* | 137,385 |
| 5 | MK780032 | *Gentiana waltonii* | 148,705 |
| 6 | KJ676538 | *Gentiana crassicaulis* | 148,776 |
| 7 | KT159969 | *Gentiana robusta* | 148,911 |
| 8 | MW316707 | *Gentiana lhassica* | 148,991 |
| 9 | MT062861 | *Gentiana manshurica* | 149,185 |
| 10 | MK602170 | *Gentiana urnula* | 149,064 |
| 11 | MZ242223 | *Gentiana szechenyii* | 149,344 |
| 12 | MG192309 | *Gentiana stipitata* | 147,156 |
| 13 | MN822304 | *Gentiana rhodantha* | 148,967 |
| 14 | MG192310 | *Gentiana veitchiorum* | 137,467 |
| 15 | MG192307 | *Gentiana oreodoxa* | 137,403 |
| 16 | MG192305 | *Gentiana hexaphylla* | 137,423 |
| 17 | MG192304 | *Gentiana caelestis* | 137,644 |
| 18 | MG192306 | *Gentiana obconica* | 137,403 |
| 19 | KX096882 | *Gentiana lawrencei* var*. farreri* | 138,750 |
| 20 | MN463101 | *Gentiana delavayi* | 149,040 |
| 21 | MN199140 | *Gentiana yunnanensis* | 147,461 |
| 22 | MT228725 | *Gentianopsis paludosa* | 151,308 |
| 23 | MT591268 | *Gentianopsis grandis* | 151,271 |
