# Supplementary material for: Comparison of plastid genomes and ITS of two sister species in Gentiana and a discussion on potential threats for the endangered species from hybridization
Source: BMC Plant Biol. 2023 Feb 20;23:101. doi: 10.1186/s12870-023-04088-z (PMC9940437; doi:10.1186/s12870-023-04088-z)
Supplement: Supplementary file 5 — Additional file 5: Table S3. The nrITS sequences of Gentiana species downloaded from NCBI. [file 12870_2023_4088_MOESM5_ESM.docx]

| **Table S3** The nrITS sequences of *Gentiana* species downloaded from NCBI | | |
| --- | --- | --- |
| No. | GenBank accession number | Species |
| 1 | KU512360 | *Gentiana tongolensis* |
| 2 | AY858678 | *Gentiana atropurpurea* |
| 3 | MT914293 | *Gentiana yunnanensis* |
| 4 | KT907724 | *Gentiana suborbisepala* |
| 5 | KT907693 | *Gentiana praeclara* |
| 6 | KT907665 | *Gentiana lineolata* |
| 7 | KT907612 | *Gentiana aristata* |
| 8 | KT907613 | *Gentiana asterocalyx* |
| 9 | KT907618 | *Gentiana bella* |
| 10 | KT907728 | *Gentiana tatsienensis* |
| 11 | KT907610 | *Gentiana aquatica* |
| 12 | KT907691 | *Gentiana piasezkii* |
| 13 | KT907609 | *Gentiana anisostemon* |
| 14 | MN339944 | *Gentiana stipitata* |
| 15 | KT907726 | *Gentiana szechenyii* |
| 16 | DQ398643 | *Gentiana robusta* |
| 17 | DQ398626 | *Gentiana waltonii* |
| 18 | MN396331 | *Gentiana lhassica* |
| 19 | MF981261 | *Gentiana crassicaulis* |
| 20 | GU251032 | *Gentiana rhodantha* |
| 21 | AY858674 | *Gentiana leptoclada* |
| 22 | GU251031 | *Gentiana primuliflora* |
| 23 | KT907637 | *Gentiana davidii* |
| 24 | AY858677 | *Gentiana veitchiorum* |
| 25 | KT907624 | *Gentiana caelestis* |
| 26 | KT907606 | *Gentiana amplicrater* |
| 27 | GU251026 | *Gentiana depressa* |
| 28 | KT907661 | *Gentiana lacerulata* |
| 29 | KT907652 | *Gentiana handeliana* |
| 30 | KT907723 | *Gentiana striolata* |
| 31 | KT907680 | *Gentiana nubigena* |
| 32 | KU512333 | *Gentiana melandriifolia* |
| 33 | KC861362 | *Gentianopsis grandis* |
| 34 | MH808532 | *Gentianopsis paludosa* |
